# Psoriasis and psychiatric disorders

**DOI:** 10.1192/j.eurpsy.2021.1716

**Published:** 2021-08-13

**Authors:** H. Ghabi, A. Aissa, S. Meddouri, U. Ouali, F. Nacef

**Affiliations:** Psychiatry A, Razi Hospital, Manouba, Tunisia

**Keywords:** psoriasis, Psychiatric comorbidities

## Abstract

**Introduction:**

Psoriasis is a common psychophysiological chronic skin disease with an important impact on patient’s quality of life. The prevalence of psychiatric conditions in psoriasis may range from 24% to 90%. The mechanisms that may explain this relationship still remain debatable.

**Objectives:**

The purpose of this work was to report two cases of psychiatric comorbidities associated with psoriasis and to discuss the possible etiopathogenic mechanisms behind this connection.

**Methods:**

To report two cases of psychiatric comorbidities associated with psoriasis.

**Results:**

Case1 Mr. A.K. is a 30-year-old male patient. He was admitted to our department in February 2020 for acute mania with psychotics features.the patient reported that since 2010, he was treated for psoriasis with local treatment (cortisone cream). The lesions did not grow or expand. Case2 Mr.A.B.is a 27-year-old male patient, with past history of psoriasis under local treatment. He is treated since 2019 in our department for schizophrenia.

**Conclusions:**

High levels of pro-inflammatory cytokines observed in psoriasis may in part explain the associated psychiatric disorders. The psychodermatologic approach would be beneficial for the adequate management of patients suffering from psoriasis.

**Disclosure:**

No significant relationships.

